# Coverage, timeliness of measles immunisation and its predictors in Pakistan: an analysis of 6.2 million children enrolled in the Provincial Electronic Immunisation Registry

**DOI:** 10.1136/bmjgh-2024-016717

**Published:** 2025-03-03

**Authors:** Manaksha Memon, Danya Arif Siddiqi, Vijay Kumar Dharma, Mubarak Taighoon Shah, Sundus Iftikhar, Hamidreza Setayesh, Subhash Chandir

**Affiliations:** 1 Maternal and Child Health, IRD Pakistan, Karachi, Pakistan; 2 Maternal and Child Health, IRD Global, Singapore; 3 Department of Infectious Disease Epidemiology & Dynamics, London School of Hygiene and Tropical Medicine, London, UK; 4 Gates Foundation, London, UK

**Keywords:** Child health, Global Health, Epidemiology, Immunisation, Public Health

## Abstract

**Background:**

Measles-related morbidity and mortality persists due to suboptimal and delayed vaccination, predominantly in low- and middle-income countries where more than 95% of global measles deaths occur. We evaluated the coverage, timeliness of measles vaccination and its predictors for children aged 12–23 months in Sindh, Pakistan.

**Methods:**

We analysed immunisation data from Sindh Province's Electronic Immunisation Registry for 6.2 million children aged 12–23 months. We assessed vaccination coverage at specific ages, calculated timeliness using Expanded Programme on Immunisation-Sindh criteria and examined predictors for timely vaccination using Cox proportional hazard regression. Spatial mapping was used to identify zero-dose measles hotspots.

**Results:**

Among 6 227 450 children aged 12–23 months, 80.6% received the first measles vaccine dose and only 58.1% of those vaccinated children aged 15–23 months received the second dose. Only 36.6% and 31.4% of children received measles-1 and 2 at the recommended age range (measles-1: 270–301 days; measles-2: 453–484 days). Subnational analysis identified 26.5% of Union Councils with ≥25% measles unvaccinated children. Children of educated mothers (≥11 years) compared with uneducated mothers had a higher timely measles vaccination likelihood (measles-1: HR=1.24; 95% CI: 1.23 to 1.26; p=0.010 and measles-2: HR=1.19; 95% CI: 1.18 to 1.21; p<0.001), while children who received the last vaccination at an outreach compared with a fixed site had a lower timely measles vaccination likelihood (measles-1: HR=0.91; 95% CI: 0.90 to 0.91; p<0.001 and measles-2: HR=0.93; 95% CI: 0.93 to 0.94; p<0.001).

**Conclusion:**

Suboptimal and delayed measles vaccination coverage casts serious doubts on attaining measles elimination by 2030, as stated in the Immunisation Agenda. Continued high-level national commitment and implementation of targeted strategies are imperative to achieving global measles immunisation goals.

**WHAT IS ALREADY KNOWN ON THIS TOPIC**

- The World Health Organization (WHO) recommends achieving 95% coverage for both doses of the measles vaccine, administered first at 9–12 months and again at 15–18 months, to establish herd immunity and protect individual children from serious illness and death.
- Timely vaccination is crucial as delays can increase susceptibility among young children, reduce community herd immunity, and potentially lead to outbreaks even where most are vaccinated.
- Understanding the factors associated with timely coverage for both doses of measles vaccine will facilitate immunisation programmes to develop and implement targeted interventions.

**WHAT THIS STUDY ADDS**

- Despite measles-1 and measles-2 coverage being 80.1% and 58.1%, respectively, three out of five children experienced delayed doses, indicating that poor timeliness of measles doses is as critical an issue as suboptimal coverage of the measles vaccine.
- One in five children enrolled in the Provincial Electronic Immunization Registry were not vaccinated for measles, and 340 hotspots of unvaccinated children were identified, pinpointing areas at greater risk of measles outbreaks.

**HOW THIS STUDY MIGHT AFFECT RESEARCH, PRACTICE OR POLICY**

- The study demonstrates how the use of big data and geographic information system (GIS) analysis to monitor vaccination coverage and identify vulnerable hotspots can shift strategies from reactive to proactive, enabling targeted interventions to prevent future outbreaks.
- Policymakers should establish and enforce policies mandating timely reporting and follow-up on missed vaccination appointments by healthcare providers, ensuring better compliance and enhancing overall vaccination coverage.
- The study underscores the need to develop and implement policies and community programmes to close vaccination gaps and conduct research to understand and address the underlying causes of suboptimal coverage, ensuring equal vaccination opportunities for all children.

## Introduction

Measles, a highly contagious viral infection of the respiratory system, remains a leading cause of morbidity and mortality among children globally, with an estimated 136 000 deaths primarily among unvaccinated children occurring in 2022.^1^ WHO recommends a vaccination coverage of 95% or greater for two doses of measles-containing vaccine to create herd immunity. The world is far below this threshold, as only 81% of children received measles-1 (first dose of the measles vaccine) by their first birthday, while only 71% of children received measles-2 (second dose of the measles vaccine) in 2021.^2^ Moreover, the COVID-19 pandemic led to disruptions in routine immunisation services and the suspension of some planned vaccination campaigns, exacerbating the risk of measles outbreaks. Particularly in Africa and parts of Southeast Asia and South America, numerous vaccination campaigns were deferred, notably affecting measles control efforts.^3^ The WHO highlighted that the deferral of 24 vaccination campaigns across 23 countries placed over 93 million individuals at heightened risk for measles, a situation aggravated in regions already contending with suboptimal vaccination coverage.^4^ Consequently, these disruptions were linked to a substantial increase in measles-related deaths worldwide, with a 43% rise from 95 000 in the previous period to 136 200 during 2021–2022.^2^

Pakistan is among the top five countries with the largest number of children unvaccinated against measles and accounts for 65% of the total measles burden in the Eastern Mediterranean Region.^5^ Although vaccines are administered free of cost through the Expanded Programme on Immunisation (EPI), estimated pre-COVID-19 national coverage rates of measles-1 and 2 vaccines plateaued at 81% and 74%, respectively, which is far below the optimal level required to attain herd immunity.^6^ The emergence of the COVID-19 pandemic and related disruptions left over 40 million children, aged 12–23 months, unvaccinated for the measles vaccine resulting in over 10 000 measles cases,^7^ leading to 800 measles-related deaths in 2021.^8^

In Pakistan, the already suboptimal measles coverage rates are further exacerbated by delayed vaccinations: only 66% and 65% of children receive the first and second doses of measles vaccine on time, that is, within EPI recommended age range of 270–301 days for measles-1 and 453–484 days for measles-2.^9^

Currently, there is limited individual-level information on suboptimal measles coverage, timely administration and factors preventing timely vaccination. To achieve measles elimination and equitable coverage, this information is crucial, especially in vulnerable low- and middle-income country (LMIC) settings like Pakistan. We used geo-enabled child-level data from the Government’s Provincial Electronic Immunization Registry, also known as Zindagi Mehfooz Electronic Immunization Registry, to assess the coverage, timeliness and equity of measles vaccination among children aged 12–23 months in Sindh, Pakistan. By analysing factors such as full immunisation coverage, zero-dose rates and dropout rates, we sought to identify risk factors for untimely vaccination. We also conducted a preliminary exploration to correlate measles coverage rates and measles cases and outbreaks. The findings of this research will contribute to a better understanding of the immunisation landscape in Sindh and inform evidence-based strategies for improving measles control and prevention.

## Methods

### Site and population

Sindh province has an annual birth cohort of 1.9 million,^10^ representing the annual target population for EPI and a total population of 53.8 million, with a density of 381.1 people/sq. km.^11^ It comprises 6 divisions, 30 districts and 1130 Union Councils (UCs).^12^ The province’s poverty index is 0.28, ranging from 0.02 to 0.50 across districts.^13^ Immunisations are primarily administered by public services, supplemented by private clinics.^13 14^ Traditionally, fixed centres provided 60% of immunisations, but post-COVID-19, this shifted to 60% via outreach visits.^14 15^ Routine outreach involves sessions held outside immunisation centres, while enhanced outreach covers larger geographical areas.^15 16^

Sindh includes urban, rural and slum areas. The 2017 population census defined urban areas as localities with metropolitan corporation, municipal corporation, municipal committee, town committee or cantonment status.^17^ Urban areas are further divided into slums and non-slums where slums are defined based on EPI-Sindh’s classification of slum areas in Karachi and Hyderabad, where UCs having >75% population living in slum areas are classified as slum UCs.^18^ Rural areas are divided into non-remote and remote, with remote areas classified by the School Education and Literacy Department as ‘hard area UCs’ in remote coastal, desert or mountainous regions.^19^

### Vaccination schedule

Pakistan’s EPI vaccination schedule includes BCG and oral polio vaccine (OPV) at birth, three doses of pentavalent vaccine (diphtheria, pertussis, tetanus, hepatitis B, Haemophilus influenzae type b), pneumococcal conjugate vaccine and OPV at 6, 10 and 14 weeks. Infants also receive two doses of rotavirus vaccine at 6 and 10 weeks, and inactivated polio vaccine (IPV) at 14 weeks and 9 months. The schedule includes two doses of measles-rubella and typhoid conjugate vaccines (TCV) at 9 and 15 months. Additions to the EPI schedule include TCV on 1 January 2020, a second IPV dose on 3 May 2021, and the rubella vaccine on 15 November 2021 (online supplemental table 3).^20 21^

### Data source

We used immunisation records from the Provincial Electronic Immunisation Registry to determine the study outcomes. The EPI estimated annual birth cohorts (data not published) informed the proportion of enrolments into the Registry. Additionally, for the supplementary analysis, we acquired measles-related incidence and impact data from the EPI Department (data not published).

### Inclusion and exclusion criteria

Children aged 0–105 months enrolled in the Provincial Electronic Immunisation Registry as of 12 March 2023, formed the source population (n=8 166 235). Exclusions were as follows: 1 628 844 children younger than 12 months, representing 19.94% of the source population and 309 941 children with missing vaccination or enrolment data, representing 3.80% of the source population. Consequently, the final study population comprised 6 227 450 children aged 12–105 months. For our analysis, we focused on vaccination data up to the age of 23 months.

### Study design and procedure

A total of 6 227 450 children aged 12–23 months were categorised into three cohorts: children receiving the first dose, both doses and no dose of measles vaccines. In the measles-2 cohort, we included those children who were 15–23 months of age and had already received the measles-1 vaccine, that is, 5 763 752. Additionally, we used data obtained from the EPI-Sindh, covering the period from 2019 to 2022, on measles cases and associated deaths to determine correlation of these variables with measles-1 up-to-date coverage at 12 months.

### Outcome definitions

The primary outcomes were up-to-date coverage, timeliness of measles vaccination and its predictors. We defined up-to-date coverage as the proportion of children who have received their measles vaccinations at specific ages: 10, 12, 18 and 23 months, as detailed in online supplemental table 4. Vaccination timeliness was assessed using the EPI-Sindh criteria, which specified that the first measles vaccine (measles-1) should be administered between 270 and 301 days of age, and the second dose (measles-2) between 453 and 484 days. A child is considered fully vaccinated under WHO guidelines^22^ if they have received one dose of BCG, three doses each of the polio and pentavalent vaccines (excluding the polio birth dose), three doses of the pneumococcal conjugate vaccine and one dose of the measles vaccine. This definition aligns with the Pakistan Demographic Health Survey (PDHS) to ensure comparability.^9^ The dropout rate is determined by subtracting the number of children who received the last vaccine in the series from those who received the first, divided by the number who received the first vaccine and multiplied by 100 to express it as a percentage. These are calculated from the initiation to the completion of a vaccine series (eg, from BCG to measles-1). For supplementary analysis, we defined zero-dose as children who have not received any dose of the pentavalent vaccine by their first birthday, helping to identify gaps in early immunisation efforts. We explore the relationship between measles cases and deaths with the timeliness of measles-1 vaccination at 12 months to understand how timely vaccination impacts outbreak control. A comprehensive list of all variables used in this analysis, along with detailed explanations, is available in online supplemental table 1.

### Statistical analysis

For summary measures, we reported frequencies (%) for categorical data and median and IQR for continuous data. We also reported the percentage of missing entries for each variable. To assess differences in measles coverage within various groups, we used a two-sample test for proportions. We used Kendall’s tau-b correlation to calculate the relationship of measles cases and deaths with measles-1 coverage at 12 months by birth cohort and enrolment year, and also assessed this correlation for the previous birth cohort and enrolment year. We applied median regression to measure differences in vaccination age by gender, location and vaccination modality. Inverse Kaplan-Meier curves estimated coverage of measles-1 and measles-2, identifying age-specific coverage rates by gender, enrolment location (slum/non-slum, urban/rural/remote rural) and enrolment modality (fixed sites, routine outreach (RO), enhanced outreach activities (EOAs) and mobile immunisation vans (MIVs)). In our supplementary analysis, we conducted spatial mapping to overlay residential areas with the prevalence of zero-dose children—those who have not received any vaccinations—and areas with children who have not received the measles vaccine. This method allowed us to identify any geographical overlaps or distinct patterns between these two groups. Furthermore, we pinpointed specific locations with high concentrations of unvaccinated children, termed ‘hotspots’. These hotspots are crucial for directing future vaccination drives and public health planning. Drawing on the methodologies used in other published studies,^23^ factors influencing the timeliness of measles vaccination were explored using a Cox proportional hazard model, with age as the underlying timescale. The a priori list of covariates included gender,^24^ place of birth,^25^ enrolment area (urban/rural), modality of most recent vaccination (outreach/fixed),^26^ receipt of SMS reminder^27^ and maternal education.^28^ We used a forward stepwise approach for the final multivariable model selection, with gender as a lock term, specifying a p value of 0.05 for entry and 0.10 for removal to identify the model with the lowest Akaike’s information criterion score. All tests were two-sided, and statistical significance was set at 0.05. Statistical analyses were performed with Stata, release 17 (StataCorp, College Station, Texas, USA).

## Results

### Baseline characteristics

Out of 6 227 450 children aged 12–23 months, 80.6% (5 017 375/6 227 450) received the first dose of the measles vaccine. In contrast, 19.4% (1 210 075/6 227 450) remained unvaccinated against measles. For children aged 15–23 months who had received measles-1, coverage for measles-2 was 58.1% (3 349 022/5 763 752). For children with available demographic data, the majority were born at hospitals (52.7%, 830 277/1 574 950), followed by home (40.5%; 638 400/1 574 950) and maternity homes (6.8%; 106 273/1 574 950). Furthermore, two out of five mothers (39.8%; 614 586/1 544 364) were uneducated. Over half (54.4%; 3 385 470/6 227 450) of the children were enrolled from urban areas, out of which 26.9% (911 818/3 385 470) were enrolled from urban slums (table 1). Subnational analysis revealed that 26.5% (340/1285) of UCs had greater than ≥25% of measles unvaccinated children (online supplemental figure 1). Children were primarily enrolled at fixed sites (56.4%; 3 513 432/6 227 450), followed by RO (25.8%; 1 603 005/6 227 450) and EOAs (17.4%; 1 080 599/6 227 450). About one in every four caregivers (25.2%, 1 533 535/6 090 658) opted for SMS reminders for the next appointment at the time of the child’s enrolment in the S (table 1).

**Table 1:** Socio-demographic characteristics and measles-1 and 2 vaccination uptake among 12–23 (n1=6 227 450) and 15–23 (n2=5 763 752) months children enrolled in the Provincial Electronic Immunisation Registry, respectively

	Measles-1 cohort (≥12 m children who received measles-1 vaccine but not measles-2 vaccine) n=1 668 353		≥15 m children who received measles-1 and measles-2 vaccine n=3 349 022		≥12 m children who did not receive measles vaccine n=1 210 075		Total n=6 227 450	
	n	%	n	%	n	%	n	%
Sex								
Male	874 458	52.4	1 759 582	52.5	635 594	52.5	3 269 634	52.5
Female	793 895	47.6	1 589 440	47.5	574 481	47.5	2 957 816	47.5
Enrolment area								
Non-remote rural	663 476	39.8	1 527 879	45.6	336 876	27.8	2 528 231	40.6
Remote rural	81 077	4.9	200 748	6	31 924	2.6	313 749	5
Urban	923 800	55.4	1 620 395	48.4	841 275	69.5	3 385 470	54.4
Enrolment subarea								
Urban slum	252 751	27.4	374 371	23.1	284 696	33.8	911 818	26.9
Urban non-slum	671 049	72.6	1 246 024	76.9	556 579	66.2	2 473 652	73.1
Place of birth*								
Hospital	206 791	52.1	424 270	51.3	199 216	56.7	830 277	52.7
Maternity home	26 735	6.7	55 197	6.7	24 341	6.9	106 273	6.8
Home	163 101	41.1	347 400	42	127 899	36.4	638 400	40.5
Mother’s education† (years)								
0	158 589	40.7	322 719	39.8	133 278	38.8	614 586	39.8
1–5	162 714	41.8	353 972	43.6	141 977	41.3	658 663	42.7
6–8	24 679	6.3	48 563	6	25 419	7.4	98 661	6.4
9–10	28 770	7.4	57 596	7.1	29 499	8.6	115 865	7.5
≥11	14 501	3.7	28 445	3.5	13 643	4	56 589	3.7
Enrolment age^‡^ (months)								
0–1	781 038	46.8	1 667 049	49.8	650 316	53.7	3 098 403	49.8
2–3	351 915	21.1	696 126	20.8	276 289	22.8	1 324 330	21.3
4–6	180 695	10.8	351 066	10.5	159 724	13.2	691 485	11.1
7–9	124 689	7.5	222 210	6.6	66 815	5.5	413 714	6.6
10–12	130 151	7.8	196 034	5.9	26 370	2.2	352 555	5.7
13–15	40 923	2.5	90 329	2.7	9194	0.8	140 446	2.3
16–18	28 827	1.7	86 078	2.6	6116	0.5	121 021	1.9
19–21	15 211	0.9	29 870	0.9	3208	0.3	48 289	0.8
≥22	14 904	0.9	10 260	0.3	12 043	1	37 207	0.6
Age at vaccination (months)	Median	IQR§	Median	IQR§	Median	IQR§	Median	IQR§
BCG/OPV-0	0.7	0.2–2.1	0.6	0.2–1.9	0.5	0.2–1.6	0.6	0.2–2.0
Penta-1/OPV-1/PCV-1	2.3	1.7–4.1	2	1.6–3.2	2.4	1.7–4.3	2.1	1.6–3.6
Penta-2/OPV-2/PCV-2	4.2	3.1–7.2	3.8	2.9–6.2	4.2	3.1–6.9	4	3.0–6.5
Penta-3/OPV-3/PCV-3	7.1	4.7–11.0	6	4.3–9.5	6	4.4–8.7	6.3	4.4–10.0
Measles-1	10.9	9.6–14.2	10.1	9.3–11.9	–	–	10.3	9.3–12.5
Measles-2	–	–	16.4	15.4–18.2	–	–	16.4	15.4–18.2
Measles-1 modality¶								
Fixed	591 642	36	1 105 461	35.5	–	–	1 697 103	35.7
Routine outreach (RO)	732 168	44.5	1 176 818	37.8	–	–	1 908 986	40.1
Enhanced outreach activities (EOAs)	308 605	18.8	824 093	26.5	–	–	1 132 698	23.8
Mobile immunisation vans (MIVs)	12 734	0.8	9000	0.3	–	–	21 734	0.5
Measles-2 modality**								
Fixed	–	–	1 067 648	31.9	–	–	1 067 648	31.9
RO	–	–	1 463 474	43.8	–	–	1 463 474	43.8
EOAs	–	–	801 665	24	–	–	801 665	24
MIVs	–	–	11 673	0.4	–	–	11 673	0.4
Provision of CNIC Numbers								
Provided	143 702	8.6	312 519	9.3	124 779	10.3	581 000	9.3
Not provided	1 524 651	91.4	3 036 503	90.7	1 085 296	89.7	5 646 450	90.7
Provision of contact numbers								
Provided	513 129	30.8	1 016 903	30.4	414 399	34.3	1 944 431	31.2
Not provided	1 155 224	69.2	2 332 119	69.6	795 676	65.8	4 283 019	68.8
SMS reminder for measles-1								
Received	100 099	6	302 378	9	125 188	10.4	527 665	8.5
Not received	1 568 254	94	3 046 644	91	1 084 887	89.7	5 699 785	91.5
SMS reminder for measles-2								
Received	–	–	207 191	6.2	67	0	289 329	4.7
Not received	–	–	3 141 831	93.8	1 210 008	100	5 938 121	95.4

* 74.7% observations for place of birth are missing in total, 76.2% observations missing for measles-1 cohort, 75.3% for children who received measles-1 and measles-2 vaccination and 71.0% for children who did not receive any dose of measles vaccine. *0.03% observations for modality are missing in total, 0.01% observations missing for measles-1 cohort, 0.01% for children who received measles-1 and measles-2 vaccination and 0.08% for children who did not receive any dose of measles vaccine.

† 75.2% observations for mother’s education are missing, 76.7% observations missing for measles-1 cohort, 75.8% for children who received measles-1 and measles-2 vaccination and 71.6% for children who did not receive any dose of measles vaccine*.*

‡ 2.2% observations for approved reminders are missing, 0.3% observations missing for children who received Measles-1 only, 3.9% who received Measles-1 and 3.9% Measles-2 vaccination and 0.1% observations missing for children who did not receive any dose of Measles vaccine.

§ Interquartile Range (75%-25%)

¶ 5.1% observations for Measles-1 vaccination modality are missing, 1.4% observations missing for Measles-1 cohort, 7.0% observations missing for children who received Measles-1 and Measles-2 vaccination.

** 0.14% observations for Measles-2 vaccination modality are missing, 0.14% observations missing for children who received Measles-1 and Measles-2.

OPVoral polio vaccinePCVpneumococcal conjugate vaccinePentaPentavalent vaccine, including vaccines against diphtheria, tetanus, pertussis, hepatitis B and Haemophilus influenza

### Up-to-date coverage, dropout rates, coverage and timeliness for measles vaccination

65.3% of children due for measles-1 were not covered up until 10 months of age, and 57.1% of children were not covered for measles-2 up until 18 months of age, indicating vaccine delays (table 2).

**Table 2:** Up-to-date cumulative immunisation coverage of 12–23 months children enrolled in the Provincial Electronic Immunisation Registry (n=6 227 450)

Antigen	Vaccination age (months)*							
	10		12		18		23	
	Due	Covered	Due	Covered	Due	Covered	Due	Covered
	n†	%	n†	%	n†	%	n†	%
BCG	5 492 307	95.4	5 492 307	97.2	4 536 551	98.4	3 932 979	98.8
OPV-0	3 555 910	99.6	3 555 910	99.7	3 017 083	99.9	2 634 327	99.9
Penta-1	5 776 724	89.7	5 792 373	92.0	4 604 013	94.1	4 012 022	94.7
OPV-1	5 774 714	89.7	5 790 558	92.0	4 601 011	94.1	4 008 878	94.7
PCV-1	5 774 543	89.7	5 790 335	92.0	4 602 042	94.1	4 010 213	94.7
Rota-1	5 613 298	86.6	5 624 920	88.9	4 266 717	90.4	3 674 533	90.6
Penta-2	5 480 368	81.4	5 495 355	85.5	4 195 941	90.6	3 697 075	92.3
OPV-2	5 478 153	81.4	5 493 293	85.5	4 194 779	90.6	3 695 673	92.4
PCV-2	5 478 244	81.5	5 493 336	85.5	4 194 830	90.6	3 695 991	92.4
Rota-2	5 021 649	79.3	5 021 649	83.4	3 678 201	88.4	3 190 849	90.0
Penta-3	5 249 362	71.1	5 249 362	78.1	3 879 379	87.9	3 493 083	92.1
OPV-3	5 248 999	71.1	5 248 999	78.1	3 879 445	87.9	3 493 235	92.1
PCV-3	5 248 551	71.1	5 248 551	78.1	3 878 995	87.9	3 492 768	92.1
IPV	5 875 059	68.4	5 895 285	74.8	4 113 448	82.7	3 674 636	85.6
Measles-1	6 226 372	34.7	6 227 450	57.6	3 950 640	74.9	3 648 821	79.8
Measles-2	–	–	–	–	2 261 045	42.9	2 687 340	58.8
FIC (without PCV)	–	–	6 227 450	45.5	3 120 599	59.2	2 877 395	63.0
FIC (with PCV)	–	–	6 227 450	45.5	3 118 569	59.1	2 875 503	62.9
FIC (with Rota)	–	–	6 227 450	40.9	2 759 575	52.3	2 507 114	54.9

* Analysis includes children who either received the vaccination in real-time or received it retrospectively but shared the vaccination date at the next follow-up visit (vaccination dates known).

† n here refers to the total number of children above a given age, and as such, represents the denominator used to calculate the coverages*.*

FICfully immunised child. Children were considered 'fully immunized' when they had received one dose of BCG, three doses of diphtheria, pertussis, tetanus, three doses of polio, and one dose of measles vaccineIPVinactivated polio vaccineOPVoral polio vaccinePCVpneumococcal conjugate vaccinePentapentavalent vaccine, including vaccines against diphtheria, tetanus, pertussis, hepatitis B and Haemophilus influenzaRotarotavirus

Considerably higher dropout rates were observed between BCG to measles-2 (38.0%) and measles-1 to measles-2 (26.2%) as compared to other vaccine intervals, that is, BCG to measles-1 (14.5%), penta-1 to measles-1 (12.1%) and penta-3 to measles-1 (5.9%) (table 3).

**Table 3:** Dropout rates among 12–23 months children enrolled in the Provincial Electronic Immunisation Registry (n=6 227 450)

Vaccine	First vaccine	Last vaccine	Dropout*	
	a	b	a−b	(a−b)/a
	n	n	n	%
BCG to measles-1	6 028 632	5 152 653	875 979	14.5
Penta-1 to measles-1	5 937 314	5 218 484	718 830	12.1
Penta-3 to measles-1	5 250 534	4 941 552	308 982	5.9
BCG to measles-2	5 313 885	3 293 392	2 020 493	38.0
Measles-1 to measles-2	4 538 760	3 349 022	1 189 738	26.2

* ((first vaccine − last vaccine) / first vaccine) × 100%).

**Figure 1:**
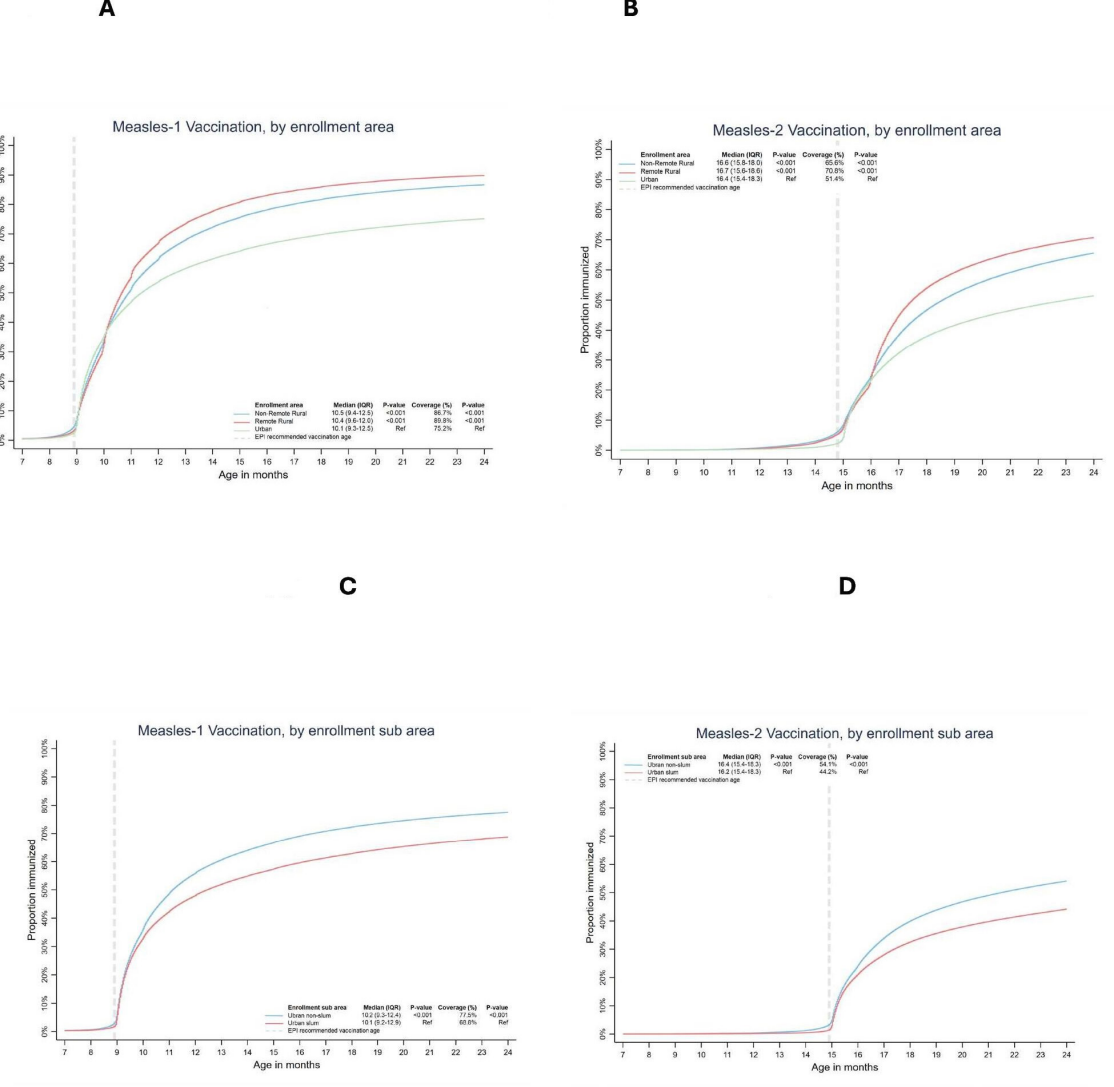
Cumulative coverage and timeliness of measles-1 (n=5 017 375) and measles-2 (n=3 349 022) among 12–23 and 15–23 months children enrolled in the Provincial Electronic Immunisation Registry by mode of delivery, gender and geography (rural, urban, slums, remote rural).

Among the children vaccinated against measles-1, 36.6% (1 834 939/5 017 375) received it on time (online supplemental table 5). The EPI recommended age for measles-1 is 9 months, whereas we found the median (IQR) vaccination age to be 10.3 (9.3–12.5) months (online supplemental figure 2 panel A). While children enrolled in remote rural areas had higher measles-1 coverage compared with urban areas (89.8% (281 825/313 749) vs 75.2% (2 544 195/3 385 470), p<0.001) (table 1), the median (IQR) age at vaccination was higher in remote rural areas (10.5 (9.4–12.5) vs 10.1 (9.3–12.5) months, p<0.001) (figure 1A). Additionally, the coverage was higher in urban non-slums as compared with urban slums (77.5% (1 917 073/2 473 652) vs 68.8% (627 122/911 818), p<0.001) (table 1) and the median (IQR) vaccination age was lower in the latter (10.2 (9.3–12.4) vs 10.1 (9.2–12.9) months, p<0.001) (figure 1C).

Furthermore, among children vaccinated with measles-2, only 31.4% (1 050 440/3 349 022) received it on time (online supplemental table 5). Our findings indicate the median (IQR) vaccination age for measles-2 to be greater than the EPI recommended age (16.5 (15.5–18.4) vs 15 months) (online supplemental figure 2 panel B). Coverage for measles-2 was remarkably higher for children enrolled in remote rural areas as compared with urban areas (70.8% vs 51.4%, p<0.001) (table 1), but the median (IQR) vaccination age was also higher, indicating delayed vaccination in remote rural areas (16.6 (15.8–18.0) vs 16.4 (15.4–18.3) months, p<0.001) (figure 1B). In urban non-slums, coverage for the second dose of measles vaccination was higher as compared with urban slums (54.1% vs 44.2%, p<0.001) (table 1) but the median vaccination age was lower in the latter (16.4 (15.4–18.3) vs 16.2 (15.4–18.3) months, p<0.001) (figure 1D). Furthermore, we did not find any significant correlation of measles cases and associated deaths with measles-1 up-to-date coverage at 12 months (online supplemental table 7.1−7.4).

### Factors associated with the timeliness of measles vaccination

For the Cox regression analysis of the timeliness of measles-1, we included complete data for all variables of 858 462 children. We found that the child’s gender had no significant impact on the timeliness of measles-1 (HR: 1.00; 95% CI: 1.00 to 1.00; p=0.990). Children born at hospitals had a 3% (HR: 1.03; 95% CI: 1.02 to 1.03; p<0.001) higher chance of receiving measles-1 on time compared with children born at home. Children enrolled in urban areas had a 4% (HR: 1.04; 95% CI: 1.04 to 1.05; p<0.001) higher likelihood while children enrolled in remote rural areas had a 3% (HR: 0.97; 95% CI: 0.96 to 0.98; p<0.001) lower chance of getting measles-1 vaccination on time as compared with children enrolled in non-remote rural areas. Children of mothers with greater than or equal to 11 years of education were 24% more likely to get vaccinated on time for measles-1 as compared with children of uneducated mothers (HR: 1.24; 95% CI: 1.23 to 1.26; p=0.010). Children who received Penta-3 vaccination through RO, EOAs and MIVs had a 9% (HR: 0.91; 95% CI: 0.90 to 0.91; p<0.001), 14% (HR: 0.86; 95% CI: 0.86 to 0.87; p<0.001) and 17% (HR: 0.83; 95% CI: 0.77 to 0.89; p<0.001), respectively, lower chance of receiving measles-1 vaccination on time than children who received Penta-3 at fixed sites. Caregivers who received SMS reminders (sent 1 day before the due date) for measles-1 vaccine were 7% (HR: 1.07, 95% CI: 1.06 to 1.08; p<0.001) more likely to have their child vaccinated timely for measles-1 compared with caregivers who did not receive SMS reminders for the vaccine (table 4).

**Table 4:** Predictors of measles-1 and measles-2 immunisation timeliness among 12–23 months children enrolled in the Provincial Electronic Immunisation Registry

	Measles-1 (n=858 462)				Measles-2 (n=650 798)			
Predictor	HR	P value	95% CI		HR	P value	95% CI	
Sex								
Male	Ref	–			Ref	–		
Female	1.00	0.987	1.00	1.00	1.00	0.759	1.00	1.01
Place of birth								
Home	Ref	–			Ref	–		
Hospital	1.03	<0.001	1.02	1.03	1.04	<0.001	1.03	1.04
Maternity home	1.01	0.070	1.00	1.02	1.02	<0.001	1.01	1.03
Urban	1.04	<0.001	1.04	1.05	1.05	<0.001	1.05	1.06
Remote rural	0.97	<0.001	0.96	0.98	0.98	<0.001	0.97	0.99
Non-remote rural	Ref	–			Ref	–		
Mother’s education (years)								
0	Ref	–			Ref	–		
1–5	1.04	<0.001	1.03	1.04	1.03	<0.001	1.02	1.04
6–8	1.05	<0.001	1.04	1.06	1.05	<0.001	1.04	1.06
9–10	1.15	<0.001	1.14	1.16	1.14	<0.001	1.13	1.16
≥11	1.24	<0.001	1.23	1.26	1.19	<0.001	1.18	1.21
Last vaccination modality, Penta-3								
Fixed	Ref	–						
Routine outreach (RO)	0.91	<0.001	0.90	0.91				
Enhanced outreach activities (EOAs)	0.86	<0.001	0.86	0.87				
Mobile immunisation vans (MIVs)	0.83	<0.001	0.77	0.89				
Measles-1 vaccination modality								
Fixed					Ref	–		
RO					0.93	<0.001	0.93	0.94
EOAs					0.91	<0.001	0.90	0.92
MIVs					0.90	0.014	0.83	0.98
SMS reminder								
Not received	Ref	–			Ref	–		
Received	1.07	<0.001	1.07	1.08	1.06	<0.001	1.05	1.07

This table presents the results of Cox regression for measles-1 and measles-2 vaccine administration from the Provincial Electronic Immunisation Registry in Sindh province, Pakistan. The outcome variable in this analysis is timeliness of receiving measles-1 and measles-2 vaccination-timely and late, where timely is receiving measles-1 vaccine from the age of 270 days to 301 days, late is receiving measles-1 vaccine after the age of 301 days; measles-2 timely vaccine from the age of 453 days to 484 days, late is receiving measles-2 vaccine after the age of 484 days*.*

76.2% data in measles-1 and 75.1% data in measles-2 analysis has been dropped, respectively, due to missing observations for mother’s education and place of birth.

Similarly, we included 650 798 children with complete data for all variables in the Cox regression analysis of the timeliness of the measles-2 vaccination. The gender variable showed no significant impact on the timeliness of the measles-2 (HR: 1.00; 95% CI: 1.00 to 1.01; p=0.759). The likelihood of receiving measles-2 on time was 4% (HR: 1.04; 95% CI: 1.03 to 1.04; p<0.001) and 2% (HR: 1.02; 95% CI: 1.01 to 1.03; p<0.001), respectively, higher among children born at hospital and maternity homes compared with those born at home. Children enrolled in urban areas had a 5% (HR: 1.05; 95% CI: 1.04 to 1.06; p<0.001) higher likelihood while children enrolled in remote rural areas had a 2% (HR: 0.98; 95% CI: 0.97 to 0.99; p<0.001) lower chance of getting measles-2 vaccination on time as compared with children enrolled in non-remote rural areas. Children of educated mothers (greater than or equal to 11 years of education) were 19% (HR: 1.19; 95% CI: 1.18 to 1.21; p<0.001) more likely to get their children vaccinated against measles-2 on time compared with children of uneducated mothers. The likelihood of timeliness of measles-2 was 7% (HR: 0.93; 95% CI: 0.93 to 0.94; p<0.001), 9% (HR: 0.91; 95% CI: 0.90 to 0.92; p<0.001) and 10% (HR: 0.90; 95% CI: 0.83 to 0.98; p=0.010) lower among children who received measles-1 vaccination through RO, EOAs and MIVs, respectively, compared with fixed sites. Caregivers who received SMS reminders (sent 1 day before the due date) for measles-2 were 6% (HR: 1.06; 95% CI: 1.05 to 1.07; p<0.001) more likely to have their child vaccinated timely for measles-2 compared with caregivers who did not receive SMS reminders for the vaccine (table 4).

## Discussion

Our study found that one in five children failed to receive any measles vaccine dose, heightening the risk of outbreaks. Additionally, three in five vaccinated children experienced delays for either dose of measles vaccine. These delays increase the period during which children are susceptible to measles. Children of less educated mothers and those enrolled through outreach programmes were more likely to experience delays. Our study reported measles-1 coverage at 80.6% and measles-2 at 58.1%, compared with the PDHS (2017–2018) rates of 73.2% and 66.6%.^9^ The differences in coverage may be due to the smaller PDHS sample size and the distinction between administrative and population-based estimates. Our analysis was based on enrolment location, capturing where the child first interacted with the system, while PDHS used residence location, potentially including migrated populations.^9^ Both sources highlight that Pakistan’s measles coverage falls short of the WHO’s≥95% benchmark for herd immunity.^2 29^ The COVID-19 lockdowns and disruptions significantly impacted immunisation services, with a 51.0% decrease in immunisation visits in Sindh, affecting measles coverage.^30^

The gap in coverage coupled with the COVID-19 pandemic strained healthcare and contributed to a global surge in measles cases and mortality, with Pakistan reporting the world’s fourth-highest measles cases in 2022. The current scenario is alarming and increases susceptibility to future measles outbreaks, as 12% of Pakistani children suffer from malnutrition. Empirical studies have shown that children with vitamin A deficiency and weak immune systems are more likely to catch measles disease than nourished populations.^31^ While our study highlights significant coverage gaps, studies have shown that administrative coverage rates may not always ensure adequate immunity.^32^ For instance, a study in Karachi found that although 90%^32^ of children were vaccinated against measles, only 55%^33^ had adequate immunity per serology tests. This underscores the need for countries to invest in surveillance and serosurveys as part of their measles elimination strategy.

Our findings revealed that about 70% of unvaccinated children for measles were enrolled in urban areas for at least one routine vaccine but did not receive either measles dose. The subnational analysis at the UC level identified 340 hotspots with a high probability of measles outbreaks. Enhanced outreach and catch-up activities in remote rural areas and urban slums successfully cover missed children, whereas urban non-slums lag in measles vaccination.

Immunisation rates for BCG and Penta 1–3 were higher compared with measles, indicated by high dropout rates: BCG to measles-1 (14.5%), Penta-1 to measles-1 (12.1%) and measles-1 to measles-2 (26.2%). These rates are higher than in other LMICs, such as India’s 8.6% BCG to measles-1 dropout rate^34^ and West Africa’s 10–20% Penta-1 to measles-1 dropout rate.^35^ Low measles coverage and high dropout rates suggest accessibility is not the main issue. EPI reports showed no measles vaccine stock-outs at the central level, indicating no systematic availability issues. Our findings point to challenges in continuing vaccination services for measles. Forgetfulness, due to the long gap between initial and measles vaccines and gaps in vaccination service organisation, where children are not effectively tracked, contribute to low measles vaccination rates.^36^ Further qualitative research on caregivers’ reasons for not returning for measles vaccines can help address these factors.

Our study revealed delays in measles vaccination, with only 36.6% and 31.4% of children receiving the measles-1 and 2 vaccines by the recommended age. This disparity is more pronounced than in the PDHS, where the difference is minimal.^9^ Delays in vaccine timeliness increase susceptibility periods, raise the risk of measles transmission and promote outbreak emergence. Despite high overall coverage, large-scale outbreaks, like those in China in 2014, occurred due to delays in timely vaccination.^37^ Timely vaccination is essential for developing herd immunity; early or late vaccinations can compromise effectiveness due to immature immune systems or interference with maternal antibodies.^38^ Studies emphasise the importance of age-appropriate vaccination to prevent measles transmission and highlight that delayed vaccination poses a significant threat.^36 39^ Evidence shows a direct correlation between untimely vaccination and outbreaks of vaccine-preventable diseases,^36 37 40^ resulting in frequent measles outbreaks in the past 4 years.^2 41^

Children of mothers with secondary or higher education were more likely to receive both measles vaccine doses on time compared with those whose mothers had primary or no education. This aligns with findings from other LMICs showing maternal education improves vaccine uptake^42^ due to increased health awareness and better health-seeking behaviour.^43^ Children enrolled in urban areas were more likely to be timely vaccinated than those in rural areas, likely because outreach activities in rural settings target delayed cases.^42 44^ Alternatively, it might also be due to caregivers delaying seeking vaccination at health facilities in anticipation of these outreach activities. Some studies and demographic surveys document better urban vaccination rates,^45^ though a study in Ethiopia found higher coverage in rural areas.^46^ Our study shows that caregivers who received SMS reminders were more likely to vaccinate their children on time. Forgetfulness, a common reason for delayed vaccination, is effectively addressed by text messaging reminders, helping caregivers track vaccination schedules and ensure timely vaccine administration.^27^

A notable strength of our study is the reliability of the Provincial Electronic Immunisation Registry data set, which uses a comprehensive system to track and monitor vaccinator activities, enhancing data accuracy. However, the study has limitations. The results are based on children enrolled in teh Registry, and while the Registry covers all public immunisation centres, it is still expanding to private centres (333 private centres used the Provincial Electronic Immunisation Registry as of 13 March 2022). Consequently, not all children vaccinated at private centres in previous years were included. Additionally, the Registry does not track doses administered during mass campaigns, meaning children who received vaccines through these campaigns might still be recorded as unvaccinated. Our analysis focuses on children with at least one interaction with the vaccination system, limiting inferences about never-immunised children who might differ from registered ones.^16^ To limit our analysis to the recommended EPI age of 12–23 months and make our findings comparable to existing literature, we applied right censoring at 23 months. However, this approach has limitations. Notably, 6.4% (399 379/6 227 450) of vaccinations in the the Registry occur beyond 23 months for either measles dose. Excluding data after 23 months might underestimate true coverage rates. Additionally, while calculating dropout rates, we did not account for deaths in the study population, potentially overestimating this measure. Our analysis of predictors of timely vaccination was limited to variables routinely collected through the Registry, excluding factors like mother’s age, occupation, household income and multiple siblings, which influence vaccination timeliness in other settings.^47^ A small proportion (3.8%) of vaccination date data was missing. These cases were excluded from the timeline analysis to ensure the accuracy of timing-related outcomes. We believe this had a negligible effect on the overall findings, as the proportion of missing data was small and any potential bias introduced is likely to be minimal. Additionally, our 6-year data analysis may include external factors like COVID-19, vaccinator strikes, heatwaves and flooding, influencing the results. Another limitation of this study is the lack of granular-level data for measles cases and deaths, which precluded detailed spatial analyses of outbreak dynamics in relation to vaccination coverage. Consequently, our findings may not fully capture the nuanced interplay between vaccination patterns and disease incidence at a localised level. Future investigations incorporating precise location-based data on measles cases and deaths would enable more in-depth analyses and help inform targeted public health interventions.

## Conclusion

We provide evidence of suboptimal measles vaccination coverage in Sindh province, Pakistan, over the past 6 years, with only 80.6% and 58.1% of children receiving the first and second doses, respectively. Vaccine timeliness is worse, with just 36.6% and 31.4% of children receiving both doses on time, contributing to the surge in measles outbreaks and related morbidity and mortality. We also identified disparities in vaccination, particularly among children of mothers with low education, residing in remote areas or urban slums and receiving vaccinations at fixed centres. Our results highlight socio-demographic and geographical factors leading to poor, delayed and inequitable measles coverage at the micro-geographical level. The findings suggest that policymakers should adopt proactive approaches to addressing poor coverage by leveraging data-driven insights, rather than relying only on reactive approaches such as post-outbreak campaigns. Integrating effective, data-driven, innovative strategies into vaccination programmes is crucial for comprehensive measles eradication.

## supplementary material

10.1136/bmjgh-2024-016717 online supplemental file 1
