# Supplementary material for: Coverage, timeliness of measles immunisation and its predictors in Pakistan: an analysis of 6.2 million children enrolled in the Provincial Electronic Immunisation Registry
Source: BMJ Glob Health. 2025 Mar 3;10(3):e016717. doi: 10.1136/bmjgh-2024-016717 (PMC11877251; doi:10.1136/bmjgh-2024-016717)
Supplement: online supplemental file 1 [file bmjgh-10-3-s001.pdf]

Supplementary Table-1: List of variables captured in SEIR and used in the study for analysis

#	Variable	Explanation	Type of variable	Options	Exogenous variables (used in analysis)
1.	Child's name	Child's full name	String		
2.	Father's name	Father's full name	String		
3.	Gender	Categorization into male and female	Categorical	Male, Female	Yes
4.	Date of birth	Child's birthdate	Date		Yes
5.	Enrollment date	Date when the child was enrolled	Date		
6.	Enrollment age	Age (days) at the time child was enrolled	Numeric		Yes
7.	Caregiver's CNIC (Optional)	National Identity Card number of the child's caregiver	Numeric		Yes
8.	Residential location	Complete residential address (optional), district, town and UC	String		Yes
9.	Mother's education (Optional)	Mother's level of education	Categorical	None, primary, middle, matric, intermediate, bachelors, masters, PHD, other	Yes
10.	Mother's language (Optional)	Language that is most commonly spoken at home (ethnicity)	Categorical	Urdu, sindhi, pashto, punjabi, saraiki, balochi, memoni , gujarati, english, bengali, hindko, other	
11.	Mother's vaccination (Optional)	Number of TT (tetanus toxoid) doses administered	Categorical	None, TT 1-5	
12.	Contact Number (Optional)	Phone number of caregiver or any other close relative	Numeric		Yes
13.	Place of delivery (Optional)	Where the child was born	Categorical	Home, Maternity Home, Hospital	Yes
14.	Enrollment location	District, town, UC and center where the child was enrolled	String		Yes
15.	Vaccination location	District, town, UC, and center where the child was vaccinated (for each vaccine)	String		
16.	Vaccination due date	Date when the child was due for a vaccine (for each vaccine)	Date		
17.	Vaccination date	Date when the child was vaccinated (for each vaccine)	Date		Yes
18.	Vaccination age	Age (days) at the time child was vaccinated (for each vaccine)	Numeric		Yes
19.	Geo-location	GPS coordinates (longitude & latitude collected at the time of enrollment and follow-up visits	Numeric		
20.	Modality	Modality through which the child was enrolled and vaccinated	Categorical	Fixed, routine outreach, EOA, mobile immunization van	Yes
21.	Vaccination status	Child's vaccination status for every vaccine	Categorical	Vaccinated, retro, retro date missing, scheduled, missing	Yes
22.	Approved SMS reminders	Consent given by the caregiver to receive SMS reminders at the time of enrollment and follow-up visits	Categorical	True, false	Yes
23.	SMS reminders schedule date	Three SMS reminders are sent for six scheduled primary vaccines (BCG, Penta 1-3 and Measles 1-2	Date		Yes
24.	SMS reminders status	The status of every SMS reminder	Categorical	Scheduled, sent, logged, canceled opted out, failed, NA and missed	Yes
**Additional variables created for analysis purposes**					
25.	Enrollment area	Categorized based on enrollment location	Categorical	Non-remote rural, remote rural, urban	Yes
26.	Enrollment sub-area	Categorized based on enrollment location of urban areas only	Categorical	Urban non-slum, urban slums	Yes
27.	Mothers' education	Categorized based on mothers' education level	Categorical	None (0), primary (1-5), secondary (6-8), matriculation (9-10), Intermediate & above (>=11)	Yes
28.	Birth year	Extracted from child's date of birth	Numeric	2014-2022	Yes
29.	Enrollment age	Categorized (in months) based on enrollment age	Categorical		Yes

#	Variable	Explanation	Type of variable	Options	Exogenous variables (used in analysis)
30.	Age at vaccination	Recalculated in months using vaccination age (days)	Numeric		Yes
31.	Timeliness	Measles timeliness were calculated using measles vaccination age (days)	Categorical	For Measles 1— Early: <270 days, Timely: 270-301 days and Delayed: >301 days<br>For Measles 2— Early: <453 days, Timely: 453-484 days and Delayed: >484 days	Yes
32.	Provision of CNIC numbers	Categorized based on the availability of CNIC numbers	Categorical	Provided, not provided	Yes
33.	Provision of contact numbers	Categorized based on the availability of contact numbers	Categorical	Provided, not provided	Yes
34.	SMS reminders	Categorized based on the caregivers' consent for receiving SMS reminders at the time of enrollment	Categorical	Opted, not opted	Yes
35.	SMS reminders for measles-1	Categorized based on the SMS reminders status for measles-1 vaccination	Categorical	Received, not received	Yes
36.	SMS reminders for measles-2	Categorized based on the SMS reminders status for measles-2 vaccination	Categorical	Received, not received	Yes
37.	M:F ratio	Calculated by dividing the number of males vaccinated for measles vaccine with number of females vaccinated for measles vaccine	Numerical		Yes
38.	Adjusted M:F ratio	Calculated by subtracting 0.055 from M;F ratio	Numerical		Yes
39.	GIR (Gender inequality ratio)	Calculated by dividing the the proportion of vaccinated boys among those who were due for vaccination by the proportion of vaccinated girls who were due for vaccination	Numerical		Yes
40.	UC range	Minimum and maximum M:F ratio/GIR among all UCs of Sindh	Numerical		Yes
41.	FIC	Received one dose of BCG, three doses of the polio vaccine (excluding birth dose), three doses of the pentavalent vaccine, three doses of pneumococcal conjugate vaccine, and one dose of the measles vaccine.	Categorical	Yes, no	Yes
42.	Drop-out rate	((first vaccine - last vaccine) ÷ first vaccine) x 100%	Categorical	Yes, no	Yes
43.	Zero-dose pentavalent	Children who failed to receive any dose of pentavalent vaccine by their first birthday.	Categorical	Yes, no	Yes
**Data received from EPI Sindh for supplementary analysis**					
44.	Measles cases	Individual cases with confirmed (clinically, laboratory, or epidemiologically) measles	Numerical		Yes
45.	Measles associated deaths	Deaths as a result of individual cases with confirmed (clinically, laboratory, or epidemiologically) measles in which death occurs within 30 days of rash onset and is not due to other unrelated causes e.g. a trauma or chronic disease	Numerical		Yes

Supplementary Table 2: Proportion of children enrolled in SEIR against the EPI estimated annual surviving infants, by birth cohort (2019-2020)

Birth cohort	EPI estimated annual surviving infants	Children enrolled in SEIR	
	n	n	%
2019	1,340,207	1,452,624	108.4
2020	1,638,386	1,581,348	96.5
2021	1,642,773	1,609,635	98.0
2022	1,682,569	1,789,419	106.4

Supplementary Table 3: Pakistan's routine immunization schedule

Disease	Causative Agent	Vaccine	Doses	Age of administration
Childhood TB	Bacteria	BCG	1	Soon after birth
Poliomyelitis	Virus	OPV	4	OPV 0: soon after birth<br>OPV 1: 6 weeks<br>OPV 2: 10 weeks<br>OPV 3: 14 weeks
		IPV	2	IPV 1: 14 weeks<br>IPV 2*: 9 months
Diphtheria<br>Tetanus<br>Pertussis<br>Hepatitis B<br>Hib pneumonia and meningitis	Bacteria<br>Bacteria<br>Bacteria<br>Virus<br>Bacteria	Pentavalent vaccine (DTP + Hep B + Hib)	3	Penta 1: 6 weeks<br>Penta 2: 10 weeks<br>Penta 3: 14 weeks
Measles and Rubella	Virus	Measles and Rubella**	2	Measles and Rubella 1: 9 months<br>Measles and Rubella 2: 15 months
Diarrhea due to rotavirus	Virus	Rotavirus	2	Rota 1: 6 weeks<br>Rota 2: 10 weeks
Typhoid	Bacteria	Typhoid Conjugate Vaccine***	1	Typhoid Conjugate Vaccine: 9 months

*Introduced on May 3, 2021
**Introduced on January 1, 2020
***Introduced on November 15, 2021

Supplementary Table 4: Up-to-date coverage definitions at predefined ages

Pre-defined ages	Numerator/Denominator
At 10 months	Children who received vaccination within ≤ 10 months of age
	Children of age ≥ 10 months of age
At 12 months	Children who received vaccination within ≤ 12 months of age
	Children of age ≥ 12 months of age
At 18 months	Children who received vaccination within ≤ 18 months of age
	Children of age ≥ 18 months of age
At 23 months	Children who received vaccination within ≤ 23 months of age
	Children of age ≥ 23 months of age

Please note that our study cohort only includes children greater than 12 months of age hence the denominators above will only include those children whereas the numerators will be calculated at vaccination ages in the study cohort.

Supplementary Figure 1: Geo-spatial analysis showing the proportion of Measles Zero-Dose children in Sindh Province

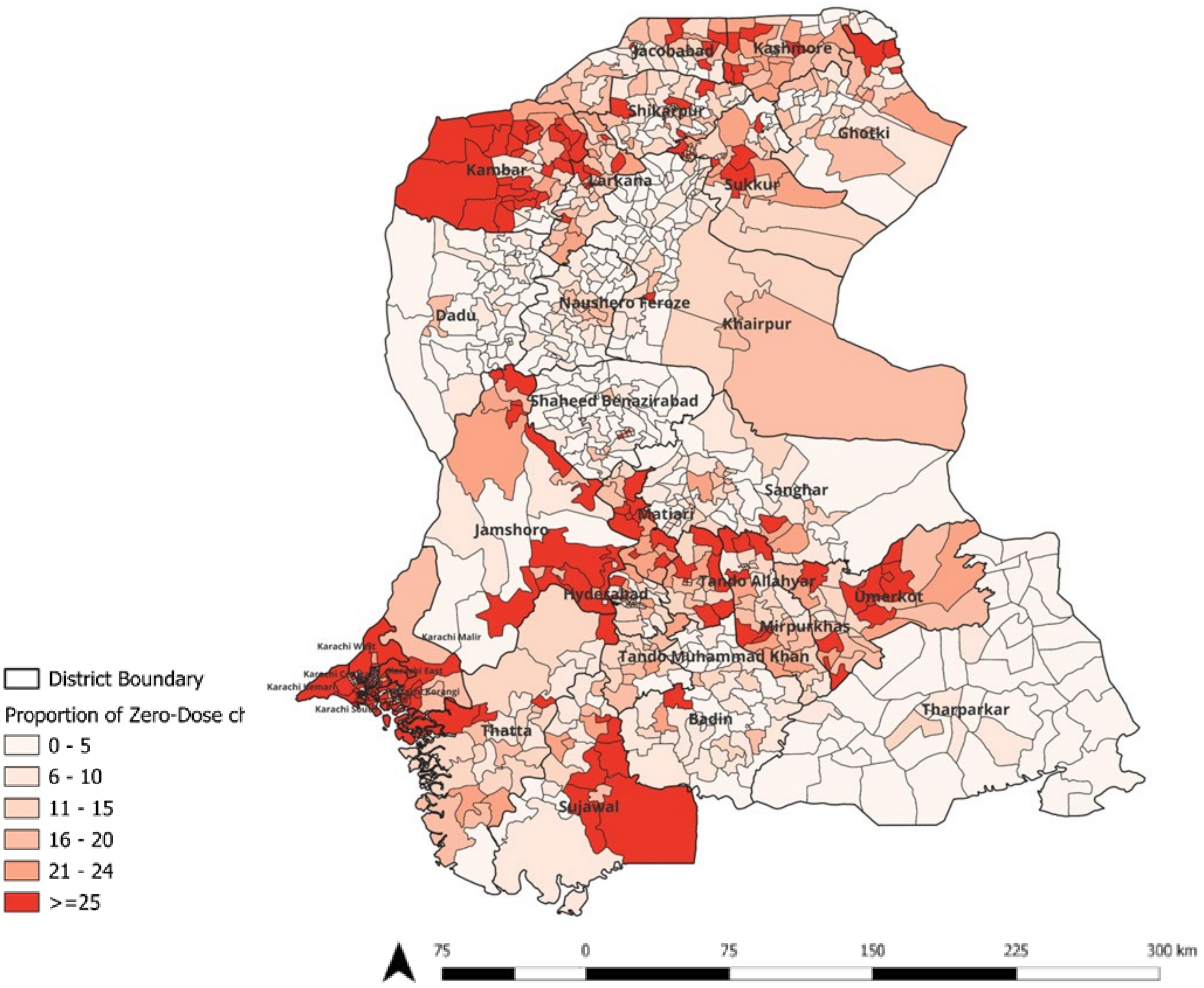

Note: 26.5% (340/1,285) of Union Councils (UCs) had greater than ≥25% of measles unvaccinated children

Supplementary Table 5: Distribution of children who received Measles vaccine by timeliness status

Timeliness	Measles-1 (n=5,017,375)		Measles-2 (n=3,349,022)	
	n	%	n	%
Early	191,756	3.8	256,948	7.7
Timely	1,834,939	36.6	1,050,440	31.4
Delayed	2,990,680	59.6	2,041,634	61.0
Total	5,017,375	100.0	3,349,022	100.0

For Measles-1— Early: <8.9 months, Timely: 8.9-9.9 months and Delayed: >9.9 months
For Measles-2— Early: <14.9 months, Timely: 14.9-15.9 months and Delayed: >15.9 months

Supplementary Table 6: Univariate analysis of predictors of Measles-1 and Measles-2 immunization timeliness among 12-23 months children enrolled in SEIR

	Measles-1				Measles-2			
**Predictor**	**Hazard Ratio**	**P-Value**	**95% Confidence Interval**		**Hazard Ratio**	**P-Value**	**95% Confidence Interval**	
**Sex**								
Male	Ref	-			Ref	-		
Female	1.00	0.250	1.00	1.00	1.00	0.740	1.00	1.00
**Place of birth**								
Home	Ref	-			Ref	-		
Hospital	1.09	<0.001	1.08	1.09	1.09	<0.001	1.09	1.10
Maternity home	1.05	<0.001	1.05	1.06	1.06	<0.001	1.05	1.07
**Enrollment area**								
Urban	1.10	<0.001	1.10	1.10	1.13	<0.001	1.13	1.13
Remote rural	0.97	<0.001	0.97	0.97	0.96	<0.001	0.96	0.96
Non-remote rural	Ref	-			Ref	-		
**Mother's education (in years)**								
0	Ref	-			Ref	-		
1-5	1.06	<0.001	1.05	1.06	1.05	<0.001	1.04	1.05
6-8	1.12	<0.001	1.11	1.13	1.11	<0.001	1.10	1.12
9-10	1.27	<0.001	1.26	1.28	1.24	<0.001	1.23	1.25
≥11	1.37	<0.001	1.36	1.39	1.30	<0.001	1.29	1.32
**Last vaccination modality, Penta-3**								
Fixed	Ref	-						
Routine outreach	0.85	<0.001	0.85	0.86				
Enhanced outreach activities	0.81	<0.001	0.80	0.81				
Mobile immunization vans	0.78	<0.001	0.76	0.79				
**Measles-1 vaccination modality**								
Fixed					Ref	-		
Routine outreach					0.87	<0.001	0.87	0.87
Enhanced outreach activities					0.82	<0.001	0.82	0.82
Mobile immunization vans					0.83	<0.001	0.82	0.85
**SMS reminder**								
Not received	Ref	-			Ref	-		
Received	1.16	<0.001	1.15	1.16	1.15	<0.001	1.15	1.16

Supplementary Table 7.1: Correlation between measles cases, deaths with up-to-date coverage at 12 months, by birth cohort

Birth cohort	Measles cases		Measles deaths	
	Correlation	P-value	Correlation	P-value
2019	-0.0112	0.3687	0.0363	0.8217
2020	-0.0589	0.6419	-0.0290	0.8608
2021	-0.0475	0.7099	0.1384	0.3199
2022	0.0444	0.7497	0.2545	0.0785

Supplementary Table 7.2: Correlation between measles cases, deaths with up-to-date coverage at 12 months, by enrollment year

Enrollment year	Measles cases		Measles deaths	
	Correlation	P-value	Correlation	P-value
2019	0.0708	0.5812	0.1181	0.4375
2020	-0.1273	0.3063	-0.0435	0.7830
2021	-0.0741	0.5559	0.1285	0.3564
2022	0.0099	0.9551	0.2489	0.0855

Supplementary Table 7.3: Correlation between measles cases, deaths with up-to-date coverage at 12 months of previous birth cohort, by birth cohort

Birth cohort	Measles cases		Measles deaths	
	Correlation	P-value	Correlation	P-value
2020	-0.1121	0.3687	0.0363	0.8217
2021	-0.0399	0.7566	0.1681	0.2257
2022	0.0247	0.8659	0.1980	0.1726

Note: We assessed the correlation of the current cohort's measles cases and deaths with the previous cohort's up-to-date coverage, as the coverage of the previous cohort can affect the occurrence of measles cases and associated deaths in the upcoming cohort.

Supplementary Table 7.4: Correlation between measles cases, deaths with up-to-date coverage at 12 months of previous enrollment year, by enrollment year

Birth cohort	Measles cases		Measles deaths	
	Correlation	P-value	Correlation	P-value
2020	0.0708	0.5812	0.1181	0.4375
2021	-0.0627	0.6199	0.1631	0.2398
2022	0.0494	0.7214	0.2206	0.1280

Note: We assessed the correlation of the current year's measles cases and deaths with the previous year's up-to-date coverage, as the coverage of the previous year can affect the occurrence of measles cases and associated deaths in the upcoming year.

Supplementary Table 8: Mean and standard deviation of children due for and vaccinated against measles vaccine per UC

# of children	Measles-1		Measles-2	
	Mean	SD	Mean	SD
Due for vaccine	5,472	4,392	5,078	4,054
Received vaccination	4,409	2,950	2,951	1,904

Figure 2: Cumulative Coverage and Timeliness of Measles-1 (n=5,017,375) and Measles-2 (n=3,349,022) among 12-23 and 15-23 months children enrolled in SEIR, overall and by sex

Panel A

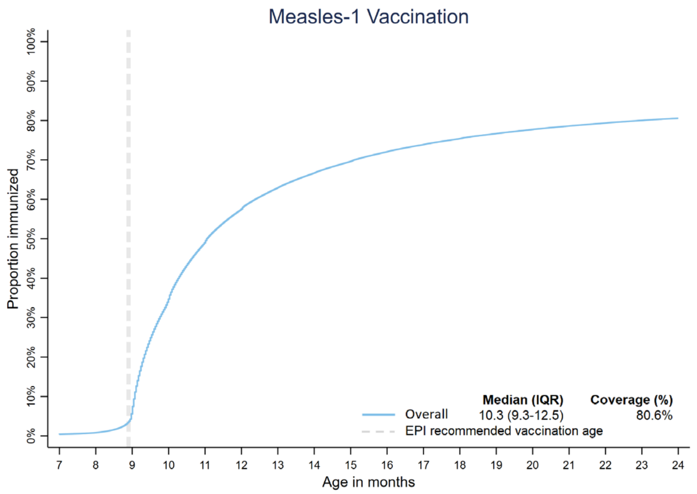

Panel B

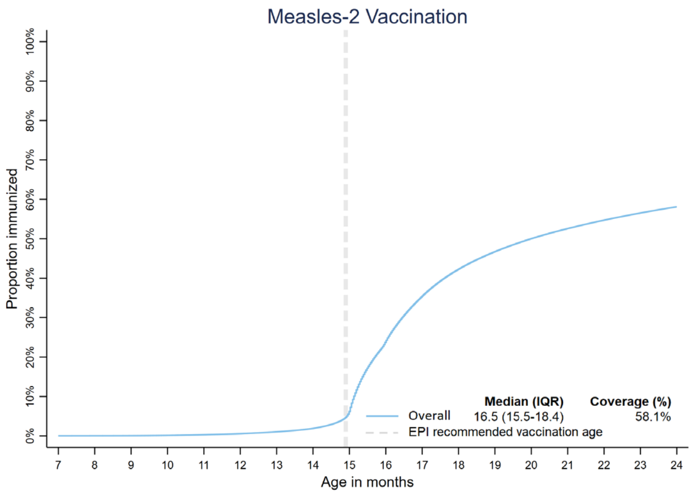

Panel C

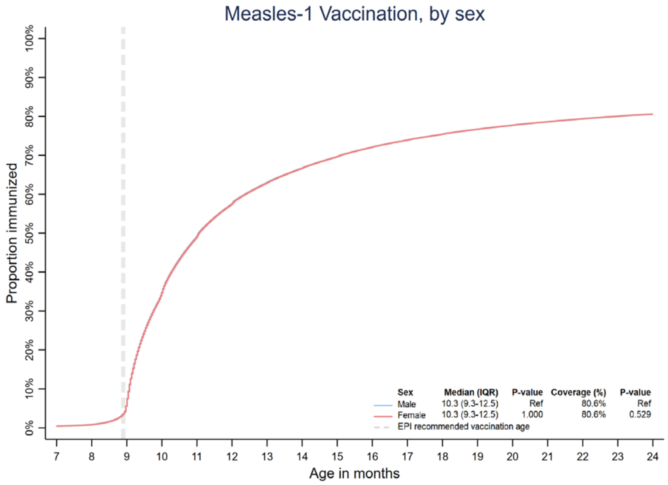

Panel D

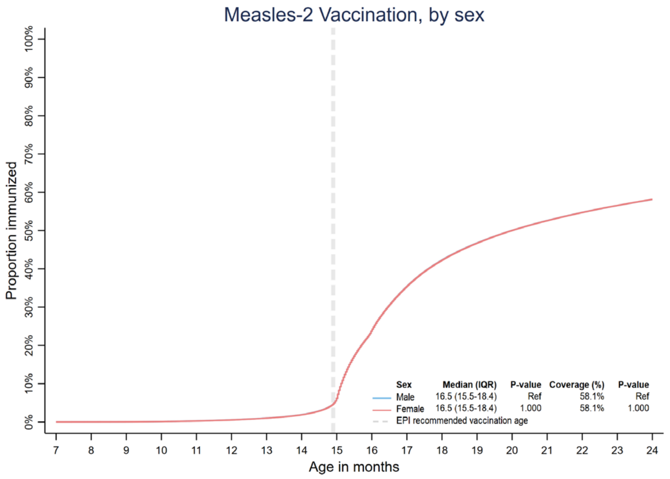

Note: For median, p-values were calculated using Median regression
For coverages, p-values were calculated using two proportions test
